# Supplementary material for: Empirical methods for controlling false positives and estimating confidence in ChIP-Seq peaks
Source: BMC Bioinformatics. 2008 Dec 5;9:523. doi: 10.1186/1471-2105-9-523 (PMC2628906; doi:10.1186/1471-2105-9-523)
Supplement: Additional File 1 — A variety of html documents from the USeq web site detailing the available applications, their best usage, output file type descriptions, command line menus, etc. [file 1471-2105-9-523-S1.zip › USeqUserGuides/JavaDoc.zip!/JavaDoc/gata/geneGlyphs/ScaleBar.html]

ScaleBar


|  |  |  |
| --- | --- | --- |
| **Overview** \| **Package** \| **Class** \| **Use** \| **Tree** \| **Deprecated** \| **Index** \| **Help** | |  |
| **PREV CLASS**   **NEXT CLASS** | **FRAMES**    **NO FRAMES**     **All Classes** |  |
| SUMMARY: NESTED \| FIELD \| CONSTR \| METHOD | DETAIL: FIELD \| CONSTR \| METHOD |  |


---


## gata.geneGlyphs Class ScaleBar

```
java.lang.Object
  gata.geneGlyphs.ScaleBar
```

---

``` public class ScaleBar extends java.lang.Object ```

**Author:**
:   Nix
    Makes a Scale bar or ruler with intervals of between 10 and 100 pixels in length at 1, 10, 100 bp or kb or mb sizes
    based on pixPerNt.

---

**Constructor Summary**

`ScaleBar(AnnoSpecParams annoParams,
GATAParams gataParams)`


| **Method Summary** | |
| --- | --- |
| `void` | `clearScaleLineArrays()`             Used in hiding scale ruler bar |
| `java.lang.String` | `getScaleBarLabel()` |
| `java.awt.geom.Line2D.Double[]` | `getScaleBarLines()` |
| `double` | `getScaleBarWidth()` |
| `java.awt.geom.Line2D.Double[]` | `getScaleLines()` |
| `java.awt.geom.Line2D.Double[]` | `getScaleRulerLines()` |
| `void` | `makeScaleBar()`             Makes a scale bar between 10 and 100 pixels in length at 1, 10, 100 bp or kb or mb sizes, change the scaleBarX and scaleBarY coordinates to relocate the bar |
| `void` | `makeScaleRuler()`             Makes a scale ruler with intervals between 10 and 100 pixels in length at 1, 10, 100 bp or kb or mb sizes, change the scaleRulerStartStopX and scaleRulerY coordinates to relocate the bar |

**Methods inherited from class java.lang.Object**

`equals, getClass, hashCode, notify, notifyAll, toString, wait, wait, wait`

**Constructor Detail**

### ScaleBar

```
public ScaleBar(AnnoSpecParams annoParams,
                GATAParams gataParams)
```


**Method Detail**

### makeScaleRuler

```
public void makeScaleRuler()
```

:   Makes a scale ruler with intervals between 10 and 100 pixels in length at 1, 10, 100 bp or kb or mb sizes,
    change the scaleRulerStartStopX and scaleRulerY coordinates to relocate the bar

---


### makeScaleBar

```
public void makeScaleBar()
```

:   Makes a scale bar between 10 and 100 pixels in length at 1, 10, 100 bp or kb or mb sizes,
    change the scaleBarX and scaleBarY coordinates to relocate the bar

---


### getScaleBarLabel

```
public java.lang.String getScaleBarLabel()
```

---


### getScaleBarWidth

```
public double getScaleBarWidth()
```

---


### getScaleBarLines

```
public java.awt.geom.Line2D.Double[] getScaleBarLines()
```

---


### getScaleRulerLines

```
public java.awt.geom.Line2D.Double[] getScaleRulerLines()
```

---


### getScaleLines

```
public java.awt.geom.Line2D.Double[] getScaleLines()
```

---


### clearScaleLineArrays

```
public void clearScaleLineArrays()
```

:   Used in hiding scale ruler bar


---


|  |  |  |
| --- | --- | --- |
| **Overview** \| **Package** \| **Class** \| **Use** \| **Tree** \| **Deprecated** \| **Index** \| **Help** | |  |
| **PREV CLASS**   **NEXT CLASS** | **FRAMES**    **NO FRAMES**     **All Classes** |  |
| SUMMARY: NESTED \| FIELD \| CONSTR \| METHOD | DETAIL: FIELD \| CONSTR \| METHOD |  |


---
